# Emerging Uses of Artificial Intelligence in Chronic Dermatologic Disease: A Scoping Review

**DOI:** 10.1177/12034754241308237

**Published:** 2024-12-30

**Authors:** Dylan Hollman, Chelsea Doktorchik, Ilya Mukovozov

**Affiliations:** 1 Department of Medicine, University of Alberta, Edmonton, AB, Canada; 2 Toronto Dermatology Centre, Toronto, ON, Canada

**Keywords:** dermatology, artificial intelligence, machine learning

## Abstract

**Background:**

Recent years have seen a surge in the use of artificial intelligence (AI) in healthcare, including dermatology. This scoping review aimed to assess the emerging applications of AI use in the context of chronic, non-neoplastic dermatologic diseases.

**Methods:**

MEDLINE, Embase, PubMed and SCOPUS were searched on August 11, 2023 using variations of the search concepts “dermatology,” “artificial intelligence,” and 12 common chronic dermatologic conditions. Article screening and data extraction were completed, and each study was categorized into themes and conditions.

**Results:**

A total of 224 unique studies were included. The most prevalent conditions that were studied in the context of AI included psoriasis (n = 67), atopic dermatitis/eczema (n = 41) and acne (n = 36). The majority of AI applications involved clinical evaluation (n = 176), images (analysis, generation or segmentation) (n = 163) and data analysis (n = 46). Clinical evaluation was further divided into 2 subthemes: diagnosis (n = 104) and disease assessment (n = 67). Diagnostic and analytic applications of AI are limited by the training datasets available (quantity of training data, image quality) and insufficient diagnostic information provided (eg, the patient’s reported history of their lesion, disease/symptom onset and risk factors).

**Conclusions:**

Common applications of AI are predominantly as an automated diagnostic tool for evaluating disease severity/characteristics, while niche and novel applications were explored further. However, recognizing the limitations of technology is critical prior to the widespread application of AI in dermatological practice. The insights from the current study can inform clinical adoption of AI in dermatology, and highlight research gaps to guide future academic initiatives.

## Introduction

In recent years, there has been an exponential increase in the development of artificial intelligence (AI) technologies. The application of these technologies to the field of healthcare is still in its infancy. AI often refers to computational technologies that have the ability to mimic or simulate the human brain in its ability to reason, think, learn, adapt, interact and understand sensory inputs.^1^ AI technology that is often employed in the field of dermatology uses deep learning (DL) paired with a neural network (NN) to assess visual inputs and provide relevant, informed outputs. One of the most common uses of this technology in a clinical setting is providing an NN with clinical images (input) that will then subsequently provide a diagnosis (output). A benefit of DL technologies is that they have the ability to independently learn pattern identification and therefore do not always require human intervention to improve the quality of their outputs.

Among medical specialties, dermatology is among the specialties at the forefront of using AI to innovate medical practice, given the large existing database of clinical images matched to specific diagnoses. An area identified as having potential is the use of this technology in dermatology as a clinical decision-support tool that would serve to increase the efficiency and accuracy of diagnostic performance by clinicians. A second, prominent use of AI within healthcare is utilizing its ability to analyze large datasets. The transition away from paper charting towards electronic medical records represents a sizeable opportunity for the analysis of patient data for purposes of epidemiologic pattern recognition. AI technologies have the ability to significantly aid in this effort.

The current study aimed to explore the ways in which AI is currently being employed as a clinical decision-making tool and for data analysis. This review also recognized novel methods currently being used in the field of dermatology that have the potential for more widespread adoption and further identified gaps in knowledge that may help guide future academic initiatives. Existing literature has focused on the application of AI to diagnose malignant and premalignant cutaneous lesions. This scoping review elected to explore the use of AI technology in the areas of non-malignant, chronic dermatologic diseases such as acne, eczema, psoriasis and beyond.

## Methods

MEDLINE, Embase, PubMed and SCOPUS were searched on August 11, 2023. Variations of the search concepts “dermatology,” “artificial intelligence,” and twelve of the most common chronic dermatologic conditions were used (Supplemental File 1). The list was adapted from epidemiologic studies showing the most common dermatologic conditions in the United States, Canada and globally.^2-5^ Variations of the term “artificial intelligence” that were used in our search include artificial intelligence, machine learning, convolution NN, DL, natural language processing, support vector machine and transfer learning, in addition to searching for existing AI technologies currently being used, such as ChatGPT, OpenAI, IBM Watson, Path AI, Covera Health and DermAssist. The 12 dermatologic conditions of focus are outlined in Supplemental File 2. We excluded studies that focused on malignant or premalignant conditions, and those studies that were technology-based but not AI. Technology systems that were encountered during our search that did not appear to be based on statistical reasoning and algorithmic learning were not deemed to be AI in nature and were not included. An example of an excludable technology would be a data processing programme used on large patient datasets that can provide summarized patient statistics but does not have the ability to improve the quality of outputs over time without human intervention or training. We only included studies that were peer-reviewed primary research, based on human data and written in the English language. Relevant titles/abstracts were exported to Covidence (Melbourne, Australia; www.covidence.org). Two reviewers independently evaluated titles and abstracts to identify relevant studies (D.H., C.D.). Disagreements were resolved through a consensus process.

Article screening and data extraction included categorizing each relevant study into themes (up to 3 per paper) and conditions (up to 2 per paper). Papers that did not have 1 to 2 exclusive conditions of focus and instead focused on multiple conditions (>2) were categorized as such “>2 Conditions of Focus.” Title, author, year and key findings were also extracted (Supplemental File 3).

## Results

After duplicates were removed, the search yielded 471 studies. Following title/abstract screening, 225 unique relevant studies were identified and results are summarized in Supplemental File 3.

### Clinical Evaluation/Image Analysis

Of the 164 studies that incorporated image analysis, all but 9 of the studies aimed to provide clinical evaluation of the suspected dermatologic condition. The subcategorization of clinical evaluation fell into 2 categories: clinical diagnosis or providing a disease assessment based on features, severity, etc. The most common use of AI is in image assessment of a cutaneous condition and providing an automated diagnostic aid tool. The reported success rates of these diagnostic AI tools vary widely and should be explored further in the subsequent analysis. In addition to providing a useful tool for automated diagnosis of skin disease, AI is also frequently being employed to assess characteristics of skin lesions, without providing a proposed diagnosis.

Thirty-three studies were focused on acne vulgaris: (19) diagnosis, (7) severity assessment, (5) classification/subtyping and (2) scar risk/classification. Zein et al used a generative adversarial network (GAN) to generate realistic artificial faces that presented with acne vulgaris.^5^ Such a model could be used for training purposes, as well as to evaluate the performance of other forms of diagnostic AI, considering that their model can produce an unlimited number of novel images. Another study used a DL model to visualize *Cutibacterium acnes* (*C. acnes*) bacteria in facial photographs that were taken with visible-light irradiation, suggesting the potential application of this technology using smartphones.^6^

Seven studies were focused on alopecia: (3) diagnosis, (3) quantifying hair loss/disease severity and (1) hair feature extraction. Shaik et al provided a case series of 3 patients receiving platelet-rich plasma treatment for androgenetic alopecia, in which they used an AI-based photography device to quantify hair fibres on the anterior, mid and vertex regions of the scalp.^7^

Twenty-six studies were focused on atopic dermatitis/eczema: (13) diagnosis, (9) severity assessment, (2) anatomical stratification and (1) subtype classification. Amongst the papers focused on the diagnostic abilities of AI, one paper developed a machine learning (ML) algorithm, in the form of an application, that was designed to automatically detect hand eczema. Their programme used a portable box housing lighting and photo equipment to allow for stable lighting conditions, limiting poor lighting and shadows, which allowed them to achieve an accuracy of 89% for the front side of the hands and 89% for the back side of the hands.^8^ Furger et al designed a GAN to generate realistic images of varying degrees and anatomical variants of eczema.^9^ This model has the potential to provide generative datasets and avoid concerns over patient privacy and image sharing.

Of the 7 studies using imaging to assess chronic wounds, (4) provided wound segmentation and only (2) for diagnosing. One study used an infrared thermography-based model paired with a convolutional neural network (CNN) to reliably detect pressure injuries 1 day before visual detection with hopes of guiding earlier prevention.^10^ Another interesting study provided a theoretical approach that uses an NN to quantify microcirculation to assess venous insufficiency, rosacea and flushing.^11^

Only 2 studies focused on hidradenitis suppurativa (HS), while the first used DL to automate a scoring tool to assess HS severity,^12^ the second study created a CNN to count and classify individual HS lesions based on images.^13^ Both studies that focused on rosacea used AI as an automated diagnostic tool, 1 study reporting an accuracy of 76% when differentiating rosacea from other similar presenting dermatoses, and an accuracy of >74% when differentiating 3 subtypes of rosacea.^14^ Similarly, all 5 studies focusing on tinea infections focused on the diagnosis of tinea; 3 of these studies focused on onychomycosis specifically.^15-17^

Five studies focused on vitiligo, with (4) using AI as a diagnostic tool and (1) using AI in disease severity assessment. The study pertaining to severity assessment used AI for a body surface area (BSA) assessment and compared the assessments to BSA done manually by dermatologists 2 months apart. Within their study, they found a higher level of accuracy and precision in AI-calculated BSA compared to dermatologists.^18^

Thirty-seven studies were focused on psoriasis: (14) diagnosis, (14) severity assessment, (5) lesion segmentation. Ahmad et al proposed an assessment method that uses 3D surface roughness with standard clustering techniques to determine the Psoriasis Area and Severity Index (PASI) score and was able to achieve an accuracy of 94%.^19^

Within the 40 studies that focused on more than 2 conditions, 3 papers stood out with novel uses of AI for dermatologic disease. The first study aimed to show the impact of race on the performance of AI in diagnosing dermatologic diseases, where they found that their AI model displayed superior diagnostic performance when trained on white skin tone images than those trained on black skin tone images.^20^ Hsu et al proposed a problem/solution that aimed to address 2 challenges to treating dermatologic disease in rural Africa: (1) the disparity in access to dermatologists between urban and rural areas, namely, in Tanzania and Madagascar, where the number of dermatologists is 1 in 2 and 3 million, respectively; and (2) the lack of data existing on African skin. Their proposed solution to this challenge was as follows: (1) since 80% of skin problems in children in their area are limited to 5 dermatoses, they would integrate AI to triage these diseases and allow local dermatologists to focus on more challenging cases through a teledermatology platform (2) begin collecting images locally to build a dataset and in the meantime, artificially increase the dataset on African skin using generative AI based on Chinese and Caucasian skin and apply these algorithms to black datasets through transfer learning.^21^ Although not a condition of focus in the current study, Mathru et al developed a novel CNN used to detect COVID-19-associated skin lesions based on clinical images. They trained their CNN with mimics of COVID-19 cutaneous manifestations and were able to achieve a sensitivity of 84% and a sensitivity of >99%.^22^

### Mobile Applications

Studies with a focus on acne vulgaris used AI technology primarily to assess the severity of the condition. Severity grading would be based on visible-light images of patient faces,^23-27^ or on estimated *C. acnes* quantity using UV light irradiation imaging.^6^ These images could be taken on smartphones and use apps employing AI to determine acne severity. One study trained the model using a validated scale, the Global Evaluation Acne, in an effort to accurately identify the number and types of lesions, in an effort to treat patients with more appropriate and tailored treatment plans.^25^

Studies with a focus on psoriasis typically used mobile applications for recognizing psoriasis, differentiating it from eczema, and discerning the severity of the disease. For example, one group of researchers trained their AI algorithm to use the PASI scoring tool to estimate the severity of psoriasis in a sample of 405 patients (1962 patients and 14,096 images to train the model). Compared to 43 dermatologist scorers, the model outperformed the dermatologists by 33%.^28^

One notable study conducted in India developed a mobile health application that used a CNN to identify 40 common skin conditions in 5014 patients with skin of colour.^29^ The model was validated using diagnoses provided by and cross-verified by a second dermatologist. The application was tested in 3 different clinical settings. Upon clinical validation, the app achieved an overall accuracy of 75%. This study set a precedent for AI-driven diagnostics in skin of colour and was further validated in a clinical setting, not only *in silico*.^29^

### Patient Sentiment and Education

Nine papers focused on assessing patient sentiment in varying capacities, the majority of which utilized language processing to analyze patient opinion. Most commonly, natural language processing (NLP) was used to analyze patient sentiment on social media forums, such as Twitter or Reddit.^30-34^ One study used various validated NLP tools such as a Relation Extraction Model, Python 3, Genism’s stop word list, Natural Language Toolkit’s stop word list and WordNet Lemmatizer, Bag of Words Model and Valence Aware Dictionary for sEntiment Reasoning in an attempt to gauge the overlying opinion of patients as positive, negative or neutral regarding disease perception or treatment options. Cummins et al assessed Reddit forums pertaining to eczema and psoriasis, and their results suggest that patients are knowledgeable about a range of treatments, including anticipated and newly approved FDA medications. Interestingly, their study also showed that patients often self-report adverse drug reactions (ADRs), highlighting the potential value of NLP to assess large data for patterns and for rare ADRs not identified during clinical trials.^30^ Similarly, Blauvelt et al used data from the IBM Watson Health MarketScan® Commercial Encounters to assess insurance claims data from the United States to determine persistence and adherence to psoriasis treatment. Their study showed that patients with psoriasis treated with ixekizumab had greater adherence to and equal or greater persistence with therapy than patients treated with guselkumab.^35^

Only 3 papers focused on patient education, 2 of which utilized ChatGPT. The first study used ChatGPT (cited as version 9) to provide a patient education guide on skin disease. This paper demonstrated that ChatGPT was able to effectively communicate at a level that could be understood by a high-school student to a newly joined college student.^36^ The second study tasked ChatGPT (version unspecified) with providing a comprehensive treatment plan, where it was able to successfully provide more information on the mechanism of action, interactions, precautions and contraindications, preparations, brand names, usage instructions, side effects and monitoring requirements than Dermnet.^37^ Lastly, the third study focused on emerging technology in the development of augmented reality that allows patients to project the different stages of development of inflammatory skin diseases, namely atopic dermatitis (AD) and psoriasis, onto their skin as holograms to visualize the onset of treatment. The authors envision a future where AI is used to enhance the knowledge base about AD and psoriasis which can then be reinforced by augmented reality for visual feedback of treatment.^38^

### Pharmacology

Thirteen papers encompassed medical pharmacotherapy as part of their focus, the majority concentrating on the use of ML towards either predicting patient response to a specified drug, or analysis of a clinical dataset for identification of ADRs.

Emam et al used ML to analyze patient variables in an attempt to predict response to biologic therapy. Their model used patient variables in 681 psoriasis patients, such as demographics, age of onset, presence of psoriatic arthritis, previous methotrexate or biologic use, dermatologic quality of life (DLQI) score and PASI scores. They were able to predict drug discontinuation with high accuracy and were able to predict treatment outcomes with <18% classification error.^39^ A similar study was able to predict psoriasis response to secukinumab using artificial NNs with an accuracy of 92% using 30 patient attributes in 23 psoriasis patients.^40^

Of the studies focusing on the identification of ADRs, the largest study used NLP (Spark NLP for Healthcare) to assess entries of physician notes in 5299 distinct dermatology patients and found 7640 instances of ADRs. Their dataset found the 5 most common causative agents were isotretinoin, spironolactone, doxycycline, tretinoin and triamcinolone, which together accounted for 17% of ADRs. By frequency, the top ADRs were rash (16%), dryness (1.9%) headaches (1.7%), joint pain (1.6%) and hair loss (1.5%).^41^ Two other notable studies that followed a similar theme included a study with a focus on ADRs caused by dupilumab in patients with AD,^42^ as well as a study identifying pharmacological classes suspected of causing skin ulcers.^43^

Turki et al used support vector machines (SVM) to identify potentially effective drugs, drug targets and therapeutic targets associated with lichen planus. Their study found 5 drugs (including, dexamethasone, retinoic acid and quercetin), 45 unique genes and 23 unique transcription factors that may be related to lichen planus pathogenesis, treatments and therapeutic targets.^44^

### Genetics and Medical Education

Foulkes et al utilized cluster sampling to analyze mRNA and small RNA transcriptome in blood, lesional and nonlesional skin to assess signals of treatment response in genes and pathways associated with TNF signaling, psoriasis pathology and major histocompatibility complex region.^45^ Another similar study developed a two-ML model of the genetic algorithm and SVM to detect relevant gene expression signatures for the classification of psoriasis.^46^ Similarly, a study out of Denmark in 2021, aimed to use 2 ML models to predict disease severity in 160 patients with AD based on measured biomarkers. They found that specific immunoinflammatory biomarkers in the serum showed a statistically significant correlation with disease severity.^47^ Lastly, Turki et al used SVM coupled with enrichment analysis to input 3 gene expression datasets pertaining to healthy and lichen planus patients with the goal of selecting important genes. Their method outperformed baseline methods in identifying disease recognition and skin tissue while identifying 45 unique genes reportedly related to lichen planus pathogenesis, treatments and therapeutic targets.^44^

Within the realm of medical education, one study used ChatGPT (cited as version 9) for the generation of educational text on skin diseases. The generated text was scored on ease and was found to be easily understood by a high-school student to a newly joined college student.^36^ Despite its present limitations, the potential use of AI-generated text to generate easily digestible patient education text on complex skin disease represents an area of immense potential in need of additional research.

### Data Analysis

In addition to clinical evaluation and image analysis being a primary use of AI technologies, analysis of large data sets has utility in all medical domains. Additional ways in which ML technology is being used towards analyzing large sets of data is through the analysis of medical records, patient surveys, medical literature, medical biomarkers and even recorded mechanical sensor data.

The ability of NLP to assess medical records has incredible potential, especially given the shift towards electronic medical record keeping in the past 5 years. One study by Seyferrt et al proposed a model by which an NLP (IBM [Armonk, New York] SPSS Statistics 25) is used to assess medical records of patients at a dermatology clinic in an attempt to identify rates and factors associated with surgical wound dehiscence (SWD) following cutaneous excisions. Their preliminary data identified a rate of SWD of 2% among 1712 instances of cutaneous excision, with all instances of SWD being significant for a current or past history of smoking. They plan to extrapolate their analysis further with more comprehensive patient data such as age, gender, anatomic location, suture method/material, immunosuppression history, anticoagulation therapy and comorbidities.^48^ Another study from 2013 sought to characterize comorbidities among vitiligo patients using NLP (model not specified). They input the medical records of 3280 patients with vitiligo and found that 23% had 1 or more comorbidities: 11% thyroid-related, 8% psoriasis, 3% rheumatoid arthritis, 2% systemic lupus, 2% inflammatory bowel disease, 2% alopecia areata and 1% type 1 diabetes mellitus. They also noted that no cases of comorbid Addison’s disease or pernicious anaemia were found.^49^

Two studies used ML to analyze skin microbiome data. Jiang et al were able to differentiate patients with AD and healthy patients based on microbiota data, as well as identifying numerous microbiota predictive of AD.^50^ Li et al used a Bayesian network to assess skin microbiome-mediated lifestyle-based environmental aggressors and found that individuals with dull, rough and acne-free skin were more exposed to sunshine and automobile exhaust, whereas individuals with dull, rough and acne skin had the highest kitchen fumes exposure.^51^

Two studies focused on using ML algorithms in conjunction with wearable mechanical sensors to detect nocturnal scratching behaviours in individuals with AD. The first study aimed to prove the effectiveness of this technology, which found a correlation of 0.96 of the algorithm’s ability to distinguish nocturnal scratching when compared to identifying scratching episodes visually through video monitoring.^52^ The second study recruited 60 healthy adult subjects who they tasked with performing a series of scratching behaviours and non-scratching behaviours while wearing the sensor. Their results showed their algorithm was able to yield a sensitivity of 92% and specificity of 98% in its ability to detect scratching behaviour.^53^

### Limitations

Limitations of AI in Dermatology are expanded on in Supplemental File 4 and include accuracy,^29^ poor classification performance,^54^ poor quality input data,^48^ poor quality images,^8^ limited available training data^5,9^ and generative/synthetic data training sets.^55^

## Discussion

Among the many explored applications of AI in the field of dermatology, clinical evaluation and image analysis were overwhelmingly the most common. The performance of these programmes is currently highly variable, but it is expected that their performance will continue to improve over time as the technology matures. Further, AI technology and subsequent applications have become increasingly innovative. Novel uses of AI have been explored in synthetic image generation for patient education and medical training purposes. These generative images also have the potential to serve as training datasets for parallel ML applications. Advanced imaging techniques have been paired with NNs for purposes of visualizing bacteria in acne, quantifying hair loss in alopecia, early visual detection of pressure wounds and quantifying disease severity in various conditions. Several studies aimed to assess the diagnostic performance differences in the skin of colour and provide solutions.

The rise of the smartphone amongst contemporary consumers provides opportunities to utilize mobile technology in conjunction with AI for a personalized clinical tool. Most commonly, mobile applications have been developed for assessing disease severity and assistance in diagnosis based on images, most often for acne, psoriasis and eczema. The evolution of the smartphone camera to capture high-resolution images has opened the door for patients to not only catalogue their disease progress but also capture up-close images that can provide specific information on lesion morphology.

Areas of focus that were understudied were genetics, dermatopathology and medical education. The studies that were found used ML to identify unique genes or patterns of gene expression that correlate to disease severity or signal treatment response. Although understudied currently, the use of generative AI, such as ChatGPT, has the potential to serve as an efficient patient education tool.

ML has the ability to analyze large data sets, such as medical records, patient surveys, medical literature, medical biomarkers and even recorded mechanical sensor data. Several studies have analyzed medical records with ML to identify rates and factors of disease severity or identify comorbidities tied to dermatologic conditions. ML has also been used to analyze patient-reported data, such as DLQI to assess disease impact on QOL. ML also has the ability to assist in the analysis of medical literature for advancing academic research. Biodata often includes incredibly large data sets which provide an opportunity for ML to identify patterns associated with specific dermatologic conditions. Wearable sensors are one such example that provide large data outputs that can lend information on scratching behaviour often seen in eczema.

The application of AI in dermatology is increasingly being used as a tool to help form a differential diagnosis or to propose a most likely diagnosis. The utility of such a tool in primary care is unproven thus far; however, the potential value can be demonstrated in a study by Federman et al which showed a performance difference of 52% versus 93% in non-dermatologists versus dermatologists, respectively, in diagnosing common skin diseases.^56^ A similar study by Scheetz et al conducted a survey of 632 ophthalmologists, radiation oncologists and dermatologists and found that 54.6% of dermatologists predicted that their respective specialty would be impacted by AI to a greater extent than that of other health professionals, such as general practitioners.^57^ Dermatologists also reported that the greatest perceived advantage of the use of AI would be improved access to disease screening. The 3 most common concerns raised regarding the use of AI were: (1) divestment of health care to large technology and data companies, (2) medical liability and (3) decreasing reliance on medical specialists for diagnosis and treatment advice. The top-ranked concern of dermatologists is the potential for medical liability due to machine error.

In conclusion, AI is most commonly used as a tool for diagnosis and disease assessment in dermatology, often utilizing image analysis for this purpose. There are numerous novel uses of AI being employed in the field that should be further explored. The impact of AI within healthcare as a whole is expected to continue to grow, as are the anticipated applications. As a field that continues to evolve, it is imperative that clinicians continue to study and test these methods thoroughly to ensure they are accurate and appropriate prior to widespread use in the field.

## Supplemental Material

sj-docx-1-cms-10.1177_12034754241308237 – Supplemental material for Emerging Uses of Artificial Intelligence in Chronic Dermatologic Disease: A Scoping Review Supplemental material, sj-docx-1-cms-10.1177_12034754241308237 for Emerging Uses of Artificial Intelligence in Chronic Dermatologic Disease: A Scoping Review by Dylan Hollman, Chelsea Doktorchik and Ilya Mukovozov in Journal of Cutaneous Medicine and Surgery

sj-docx-2-cms-10.1177_12034754241308237 – Supplemental material for Emerging Uses of Artificial Intelligence in Chronic Dermatologic Disease: A Scoping Review Supplemental material, sj-docx-2-cms-10.1177_12034754241308237 for Emerging Uses of Artificial Intelligence in Chronic Dermatologic Disease: A Scoping Review by Dylan Hollman, Chelsea Doktorchik and Ilya Mukovozov in Journal of Cutaneous Medicine and Surgery

sj-docx-4-cms-10.1177_12034754241308237 – Supplemental material for Emerging Uses of Artificial Intelligence in Chronic Dermatologic Disease: A Scoping Review Supplemental material, sj-docx-4-cms-10.1177_12034754241308237 for Emerging Uses of Artificial Intelligence in Chronic Dermatologic Disease: A Scoping Review by Dylan Hollman, Chelsea Doktorchik and Ilya Mukovozov in Journal of Cutaneous Medicine and Surgery

sj-xlsx-3-cms-10.1177_12034754241308237 – Supplemental material for Emerging Uses of Artificial Intelligence in Chronic Dermatologic Disease: A Scoping Review Supplemental material, sj-xlsx-3-cms-10.1177_12034754241308237 for Emerging Uses of Artificial Intelligence in Chronic Dermatologic Disease: A Scoping Review by Dylan Hollman, Chelsea Doktorchik and Ilya Mukovozov in Journal of Cutaneous Medicine and Surgery
