# Supplementary material for: Emerging Uses of Artificial Intelligence in Chronic Dermatologic Disease: A Scoping Review
Source: J Cutan Med Surg. 2024 Dec 30;29(3):274–81. doi: 10.1177/12034754241308237 (PMC12171080; doi:10.1177/12034754241308237)
Supplement: sj-docx-1-cms-10.1177_12034754241308237 – Supplemental material for Emerging Uses of Artificial Intelligence in Chronic Dermatologic Disease: A Scoping Review [file sj-docx-1-cms-10.1177_12034754241308237.docx]

**Supplemental File 1**

**Ovid MEDLINE(R) ALL <1946 to August 11, 2023>**

1 exp Dermatology/ 21262

2 "dermatolog*".tw. 71121

3 1 or 2 78941

4 exp Artificial Intelligence/ or exp Machine Learning/ or exp Neural Networks, Computer/ or exp Deep Learning/ or exp Natural Language Processing/ or exp Support Vector Machine/ 177006

5 ("AI" or "artificial intelligence" or "machine learning" or "deep learn*" or "neural network*" or "ChatGPT" or "OpenAI" or "IBM Watson" or "PathAI" or "Covera Health" or "DermAssist" or "natural language processing" or "support vector machine" or "transfer learning" or "representation learning").tw. 249697

6 4 or 5 325191

7 exp Dermatitis, Atopic/ or exp Eczema/ or exp Psoriasis/ or exp Rosacea/ or exp Dermatitis, Contact/ or exp Alopecia/ or exp Alopecia Areata/ or exp Vitiligo/ or exp Dermatitis, Seborrheic/ or exp Hidradenitis Suppurativa/ or exp Acne Vulgaris/ or exp Chronic Urticaria/ or exp Lichen Planus/ or exp Foot Ulcer/ or exp Leg Ulcer/ or exp Pressure Ulcer/ or exp Skin Ulcer/ or exp Diabetic Foot/ or exp Tinea/ or exp Tinea Versicolor/ 225595

8 ("atopic dermatitis" or "eczema" or "psoriasis" or "rosacea" or "contact dermatitis" or "alopecia" or "vitiligo" or "seborrheic dermatitis" or "hidradenitis suppurativa" or "acne inversa" or "acne" or "chronic urticaria" or "lichen planus" or "stasis dermatitis" or "wound*" or "ulcer*" or "tinea" or "ringworm").tw. 624698

9 7 or 8 701523

10 3 and 6 and 9 153

11 limit 10 to (english language and humans) 100

**Embase <1974 to 2023 August 11>**

1 exp dermatology/ 52133

2 "dermatolog*".tw. 127508

3 1 or 2 151214

4 exp artificial intelligence/ or exp machine learning/ or exp artificial neural network/ or exp convolution neural network/ or exp deep learning/ or exp natural language processing/ or exp support vector machine/ 441688

5 ("AI" or "artificial intelligence" or "machine learning" or "deep learn*" or "neural network*" or "ChatGPT" or "OpenAI" or "IBM Watson" or "PathAI" or "Covera Health" or "DermAssist" or "natural language processing" or "support vector machine" or "transfer learning" or "representation learning").tw. 301671

6 4 or 5 540723

7 exp atopic dermatitis/ or exp eczema/ or exp psoriasis/ or exp rosacea/ or exp contact dermatitis/ or exp alopecia/ or exp alopecia areata/ or exp vitiligo/ or exp seborrheic dermatitis/ or exp suppurativa hidradenitis/ or exp acne/ or exp acne vulgaris/ or exp chronic urticaria/ or exp lichen planus/ or exp stasis dermatitis/ or exp foot ulcer/ or exp leg ulcer/ or exp pressure ulcer/ or exp skin ulcer/ or exp diabetic foot/ or exp tinea/ or exp tinea versicolor/ 425489

8 ("atopic dermatitis" or "eczema" or "psoriasis" or "rosacea" or "contact dermatitis" or "alopecia" or "vitiligo" or "seborrheic dermatitis" or "hidradenitis suppurativa" or "acne inversa" or "acne" or "chronic urticaria" or "lichen planus" or "stasis dermatitis" or "wound*" or "ulcer*" or "tinea" or "ringworm").tw. 834939

9 7 or 8 995822

10 3 and 6 and 9 393

11 limit 10 to (english language and humans) 361

**PubMed <August 11>**

(((dermatology[MeSH Terms]) OR (dermatolog*[Title/Abstract])) AND (((ai artificial intelligence[MeSH Terms]) OR (artificial intelligence[MeSH Terms]) OR (machine learning[MeSH Terms]) OR (neural network computer[MeSH Terms]) OR (neural network model[MeSH Terms]) OR (natural language processing[MeSH Terms]) OR (support vector machine[MeSH Terms])) OR ("AI"[Title/Abstract] OR "artificial intelligence"[Title/Abstract] OR "machine learning"[Title/Abstract] OR "deep learn*"[Title/Abstract] OR "neural network*"[Title/Abstract] OR "ChatGPT"[Title/Abstract] OR "OpenAI"[Title/Abstract] OR "IBM Watson"[Title/Abstract] OR "PathAI"[Title/Abstract] OR "Covera Health"[Title/Abstract] OR "DermAssist"[Title/Abstract] OR "natural language processing"[Title/Abstract] OR "support vector machine"[Title/Abstract] OR "transfer learning"[Title/Abstract] OR "representation learning"[Title/Abstract])) AND (((atopic dermatitis[MeSH Terms]) OR (atopic eczema[MeSH Terms]) OR (psoriasis[MeSH Terms]) OR (acne rosacea[MeSH Terms]) ANORD (papulopustular rosacea[MeSH Terms]) OR (allergic contact dermatitis[MeSH Terms]) OR (alopecia[MeSH Terms]) OR (alopecia areata[MeSH Terms]) OR (vitiligo[MeSH Terms]) OR (dermatitis, seborrheic[MeSH Terms]) OR (hidradenitis suppurativa[MeSH Terms]) OR (acne inversa[MeSH Terms]) OR (acne[MeSH Terms]) OR (acne vulgaris[MeSH Terms]) OR (stasis dermatitis[MeSH Terms]) OR (lichen planus[MeSH Terms]) OR (foot ulcer[MeSH Terms]) OR (leg ulcer[MeSH Terms]) OR (pressure ulcer[MeSH Terms]) OR (skin ulcer[MeSH Terms]) OR (diabetic foot[MeSH Terms]) OR (tinea[MeSH Terms]) OR (tinea versicolor[MeSH Terms])) OR ("atopic dermatitis"[Title/Abstract] OR "eczema"[Title/Abstract] OR "psoriasis"[Title/Abstract] OR "rosacea"[Title/Abstract] OR "contact dermatitis"[Title/Abstract] OR "alopecia"[Title/Abstract] OR "vitiligo"[Title/Abstract] OR "seborrheic dermatitis"[Title/Abstract] OR "hidradenitis suppurativa"[Title/Abstract] OR "acne inversa"[Title/Abstract] OR " acne"[Title/Abstract] OR "chronic urticaria"[Title/Abstract] OR "lichen planus"[Title/Abstract] OR "stasis dermatitis"[Title/Abstract] OR "wound*"[Title/Abstract] OR "ulcer*"[Title/Abstract] OR "tinea"[Title/Abstract] OR "ringworm"[Title/Abstract])) AND (English[Language])) 149

**Scopus <August 11>**

( TITLE-ABS ( "dermatolog*" ) AND TITLE-ABS ( "AI" OR "artificial intelligence" OR "machine learning" OR "deep learn*" OR "neural network*" OR "ChatGPT" OR "OpenAI" OR "IBM Watson" OR "PathAI" OR "Covera Health" OR "DermAssist" ) AND TITLE-ABS ( "atopic dermatitis" OR "eczema" OR "psoriasis" OR "rosacea" OR "contact dermatitis" OR "alopecia" OR "vitiligo" OR "seborrheic dermatitis" OR "hidradenitis suppurativa" OR "acne inversa" OR "acne" OR "chronic urticaria" OR "lichen planus" OR "stasis dermatitis" OR "wound*" OR "ulcer*" OR "tinea" OR "ringworm" ) AND LANGUAGE ( english ) ) 195
