# Supplementary material for: Emerging Uses of Artificial Intelligence in Chronic Dermatologic Disease: A Scoping Review
Source: J Cutan Med Surg. 2024 Dec 30;29(3):274–81. doi: 10.1177/12034754241308237 (PMC12171080; doi:10.1177/12034754241308237)
Supplement: sj-docx-2-cms-10.1177_12034754241308237 – Supplemental material for Emerging Uses of Artificial Intelligence in Chronic Dermatologic Disease: A Scoping Review [file sj-docx-2-cms-10.1177_12034754241308237.docx]

**Supplement 2:** Included Papers With Associated Themes and Conditions

|  | Acne Vulgaris | Alopecia | Atopic Dermatitis | Chronic Urticaria | Chronic Wound | Contact Dermatitis | Hidradenitis Suppurativa | Lichen Planus | Psoriasis | Rosacea | Tinea Infection | Vitiligo | >2 Conditions of Focus |
| --- | --- | --- | --- | --- | --- | --- | --- | --- | --- | --- | --- | --- | --- |
| Clinical Evaluation | 32 | 7 | 29 | - | 7 | - | 2 | 2 | 44 | 5 | 6 | 4 | 52 |
| Diagnostic | 14 | 1 | 19 | - | 3 | - | - | 2 | 20 | 4 | 6 | 2 | 51 |
| Disease Assessment | 18 | 6 | 10 | - | 4 | - | 2 | - | 24 | 1 | - | 2 | 1 |
| Data Analysis | 2 | - | 12 | - | 2 | - | - | - | 21 | - | - | 2 | 13 |
| Dermatopathology | - | - | - | - | - | - | - | - | - | - | - | - | - |
| Dermoscopy | 1 | - | 1 | - | - | - | - | - | 4 | - | 1 | - | 3 |
| Ethics | - | - | - | - | - | - | - | - | - | - | - | - | - |
| Genetics | - | - | 2 | - | - | - | - | 1 | 3 | - | - | - | - |
| Images | 33 | 7 | 29 | - | 7 | - | 2 | 1 | 44 | 4 | 6 | 5 | 45 |
| Manuscript Writing | - | - | - | - | - | - | - | - | - | - | - | - | - |
| Medical Education | - | - | - | - | - | - | - | - | - | - | - | - | 1 |
| Mobile Applications | 6 | 1 | 3 | - | 1 | - | - | - | 5 | - | 1 | - | 5 |
| Patient Education | - | - | 1 | - | - | - | - | - | 1 | - | - | - | 2 |
| Patient Sentiment | 1 | - | 2 | - | - | - | - | - | 4 | - | - | - | 4 |
| Pharmacology | - | - | 1 | - | 1 | - | - | 1 | 7 | - | - | - | 2 |
| Prognosis | 2 | - | - | - | - | - | - | - | 4 | - | - | - | 1 |
| Per included paper, up to three themes and up to two conditions were identified. | | | | | | | | | | | | | |
