# Supplementary material for: Emerging Uses of Artificial Intelligence in Chronic Dermatologic Disease: A Scoping Review
Source: J Cutan Med Surg. 2024 Dec 30;29(3):274–81. doi: 10.1177/12034754241308237 (PMC12171080; doi:10.1177/12034754241308237)
Supplement: sj-docx-4-cms-10.1177_12034754241308237 – Supplemental material for Emerging Uses of Artificial Intelligence in Chronic Dermatologic Disease: A Scoping Review [file sj-docx-4-cms-10.1177_12034754241308237.docx]

**Supplement 4:** Limitations of AI in Dermatology

| **Topic** (related studies) | **Considerations and Limitations** |
| --- | --- |
| Accuracy^29^ | Accuracy of mobile application diagnostic tools can be compromised as the history of conditions, including duration of lesion, sites involved, symptoms, evolution, and distribution is often unknown. |
| Poor Classification Performance^54^ | Poor classification performance based on inappropriate or insufficient datasets used to train neural networks. |
| Poor Quality Input Data^48^ | Using big data analysis for pattern recognition, the analysis is often only as good as the input data. Input data is often unstandardized in EMR systems and requires substantial efforts to standardize the data to a point that it would be adequate for analysis. |
| Poor Quality Images^8^ | When machine learning algorithms are trained based on images, the diagnostic accuracy can be negatively impacted by poor image quality, poor lighting or the presence of shadows. |
| Limited Available Training Data^5,9^ | Machine learning is typically trained on a dataset that is pre-classified by experts in the field. The more data an AI algorithm has to learn from, the higher its accuracy generally is. Limitations exist with access to large data sets, particularly where patient permission is needed. |
| Generative/Synthetic Data Training Sets^55^ | Using synthetic or generative AI images to train neural networks comes with concerns regarding realistic application of data sets. |
